# The Mortality of Infancy

**Published:** 1871-06

**Authors:** 


### THE MORTALITY OF INFANCY.

The great mortality among infants, especially in our large cities, ought to attract more attention than it receives—not from the medical profession alone, but from the philanthropic public generally. Does not this mortality result in part from causes which may be removed, at least to some extent, is a question to be considered, not only by the medical man, but the political economist. The mortality among infants is much greater in the cities than in the country, and very much greater among the poor than among the wealthy, It is well known, also, that the mortality among the infants of our colored population, is far greater since the emancipation than before. These facts prove, if proof were needed, that this mortality is caused to a great extent by want, bad air. ignorance, and the vices attendant upon poverty. These bad influences can to some extent be removed.

Those parts of the city where the poor are crowded together, ought to be kept as cleanly, the sewerage should be as thorough, the water supply as abundant, as in the more wealthy parts of the city, albeit the taxes accruing therefrom are not so great.

Economy itself dictates this. The poor are a burden upon the rich. This burden might be greatly lessened by a policy having for its object the prevention of suffering, as well as the relief thereof. We respectfully suggest that if benevolent societies would build substantial, well-ventillated houses, and rent them at low rates, or even charge no rent, reserving and thoroughly exercising the right of inspection as to cleanliness, the burden upon the charitable would be lessened, and the amount of suffering greatly diminished.

The infant has a struggle for life in this rough world at best, but when the mother, during gestation, is suffering for the very necessaries of life, her mind constantly harassed by the one absorbing question, "what shall I eat and wherewithal shall I be clothed" living in filth, not even breathing the pure, fresh air of Heaven, but a poisonous atmosphere, freighted with disease and death, it is not surprising that the child comes into the world illy prepared for life's battle, even if placed immediately after birth under the best influcnce. But subject to the same circumstances which surround the mother—subjected to the mismanagements and imprudencies incident to ignorance, and dependant for nourishment upon a mother, whose physical condition renders it impossible for her to furnish nutriment of proper character or in sufficient amount; the child soon sickens and dies, or if it lives, drags out a miserable existence, the constant prey to suffering and disease.

But it is not the child of want only who suffers from insufficiency of food. Whether or not it is one of the unavoidable results incident to civilization, it seems to be a fact indisputably true that modern mothers have not the same development of the lacteal organ possessed by the mothers of old. Neither is it the soft flabby breast alone that we find this defect, but it often exists where the organ is apparently well-developed. It has been estimated that not more than one-half of the mothers of the present day afford milk enough for their offspring. In very many cases this is not appreciated. The child becomes emaciated, is fretful and sleepless, suffers from colic, etc., etc. Medicine is given—stimulants and anodynes, while the cause of the whole trouble is starvation. This is very frequently the case, also, when the infant is fed by hand, for we believe the amount of food required by an infant is much greater than is generally supposed. In very many cases, their food is poured into their mouths from a spoon with difficulty, and in small quantity. "Babies' food" compounds, frequently of doubtful utility, at least if milk is used; it is a very little milk with a great deal of water. In such cases the bottle should be always used and the milk should be, by proper modification, made to resemble as nearly as possible the mother's milk. We agree with Dr. W. M. Welch, who says in an article in the Med. Times, that "by referring to a table of analyses of milk, we see in asses' milk the closest resemblance; but as this can rarely be procured, cows' milk, on account of the facility with which it can always be obtained, is the one to which recourse is usually had. It is important, too, that the milk should be taken from a healthy cow, at liberty to feed and graze at pleasure, (not stall-fed), and, if possible, always from the same animal; because, as Dr. Dewees remarks, "different cows feeding upon the same materials often give different qualities of milk, and the stomach very generally becomes reconciled more easily to any one certain quality than to a mixture."

The milk of the cow, being intended to meet the wants of a strong and vigorous animal, cannot be rationally administered to a young and delicate infant, of much feebler digestive powers, without first undergoing some modification.

The difference between human milk and cows' milk, as ascertained by analysis, is as follows: Human milk contains casein, 32, sugar, 36, butter 29; cows' milk, casein, 63, sugar 28, butter 40. It will be seen from this that if cows' milk be reduced one half, the casein will be about the same as in human milk, the butter slightly less, and the sugar only one-third of what it should be. In adapting it, therefore, to an infant, the first six or eight weeks it should be diluted with equal parts of water, have added a little sweet cream, and must be sweetened by adding to each six ounces of the mixture about one-half teaspoonful of sugar of milk, or lump sugar; the ordinary brown sugar should never be used, as it contains material which will more readily decompose and give rise to fermentation. After six or eight weeks the dilution need not be so great; one-third water will probably be sufficient. After three or four months the quantity of water may be still further reduced, say one-fourth water to three-fourths milk. And after six months the milk may be given undiluted. During all this time the quantity of sugar and cream should remain the same. Any change in the dilution of the milk must be made with great care; for we must recollect that we can at the best but poorly imitate Nature in her increase of the casein in proportion to the growing wants of the child.

The rule which I have given will not apply to the milk which is served from door to door in large cities. Indeed, no rule will apply to such milk, for it is always of uncertain quality. Each case must determine for itself the degree of dilution, if any, and the mode of preparation.

The intervals of feeding should be regular, and the quantity given should also be carefully regulated. For the first two or three weeks, three or four fluid ounces every two or three hours will, generally speaking, be sufficient. As the child grows older, this quantity must be gradually increased. At this rate it will be seen that the child is getting in twenty-four hours at least a pint of pure cow's milk, which ought to be sufficient, if properly digested, to meet its wants at this early age.

The mode of administering the milk is also important. It should always be given in imitation of Nature's way, by sucking. A child will almost always instinctively suck from its earliest age. By this means it swallows the milk very gradually, and is, therefore, less apt to overload its stomach. The first show of indifference on its part is a sure sign that it has enough, and it should never be pressed to continue. When it has finished its meal, the bottle should be at once removed; for if permitted to suck at an empty bottle it will swallow air, which will give rise to colic pains. If any milk remains in the bottle, it should be at once emptied out, and not kept for a subsequent feeding, as it is liable to undergo fermentation. The bottle and all parts connected with it should then be well cleansed, and placed in water until required again. If perfect cleanliness be not scrupulously observed, the bottle will soon smell sour, showing that some milk has been left to ferment. Fresh milk added while this remains will turn sour in a very short time. The sugar and water should not be added until the meal is required. The mixture should then be gradually raised to a temperature of 95 to 98 degrees. Should any tendency to acidity be observed in the milk, it should be rejected forthwith. No attempt at its supposed restoration should be made, by the addition of sugar or other agents, as these will eventually but increase the evil.

These precautions may seem minute, and, to some unnecessary; but their importance cannot be overestimated. In proportion as they are deviated from, the risks to the child's life increase. They may also involve some little care on the part of the mother, whose duty it should be to supervise the preparation of the food; but she will be abundantly compensated for her trouble by securing what is most to be desired, her child's life and health.

The farinaceous articles are highly improper as food for very young infants, for three reasons,—viz.:

1. Because of their inability to digest them. The conversion of tarsch into glucose, or grape-sugar, is begun by the saliva and completed by the intestinal juices. Now, the saliva is not secreted in the infant before the fourth month, nor does the intestinal juice of a very young infant seem to have the power of converting starch into grape sugar, as would appear from the fact that in post mortem examinations of children who during their lifetime had been largely fed on farinaceous articles, a starchy film has been found lining the intestines, which yielded the charaateristic blue color to the iodine test.

2. They do not contain the four classes of food in the proportion required for healthy nutrition,—viz: albumen, fatty substances, carbo-hydrates, and salts; all of which are contained in milk, in the form of casein, butter, sugar and salts.

3. Supposing them to be digested, starches, and sugars into which starches are converted, have a greater affinity for oxygen than the albuminates have; they therefore tend to appropriate the oxygen which is required to combine with the waste tissues in order to effect their elimination, and they thus impede the proper nutritional changes; or, in other words, they are heat-giving rather than tissue-making materials.

While we regard the milk of an animal as the best substitute which can be furnished to a child in lieu of its mother's milk, it must not be forgotten that it can serve at the best only as a substitute, for it is not, and cannot be made, identical. It would seem, as every variety is composed of the same constituents, only varying in their relative proportions, to be an easy matter to balance these differences and thus make them identical. But it is not so. These constituents have different properties. Take, for instance, human milk and the milk of the cow. Examine their chief nutritive constituent—casein. It will be found, if rennet be added to human milk, that its casein will coagulate into light loose clots, formed by the aggregation of little flocculi, which offer no impediment to the feeble digestive powers of the infant, on account of being the most easily digested of all known articles. On the other hand, add rennet to cow's milk, and its casein will coagulate into heavy compact lumps. The same thing takes place within the child's stomach, as may be seen by observing the milk vomited shortly after feeding. We have seen these lumps so large and tough as almost to choke the child when in the act of vomiting them, and have even found it necessary to assist in their removal from its mouth and fauces. They also may frequently be found in its passages, and even in a large and compact mass in its stomach after death.

This heavy, dense clot, then, of cow's milk, is more difficult of digestion, and taxes the feeble digestive powers of the young infant to their utmost, which even then are often unequal to the task, permitting some of the casein to remain undigested, which, if not thrown off by vomiting, will speedily undergo fermentation, and give rise to acidity and diarrhœa, and, if the error of diet be continued, may lead to more serious results.

It must be remembered that it is not that which is taken into the stomach that nourishes, but only that which is digested. Many a mother or nurse, ignorant of this fact, seeing that her child artificially fed does not grow properly, or it may be, is losing its plumpness, will, therefore, infer that something more solid is required. With this view she resorts to one or more of the many vile farinaceous compounds prepared and sold in shops under the name of "food for infants." But, alas for the poor victim of misdirected sympathies! this but adds distress to its discomfort, which if the error of diet be not corrected, will continue to increase until some kind disease intervenes to relieve it of its suffering.

Rather let mothers and nurses be taught that digestion is essential to developmeht, and that without it a child may actually starve on the fullest diet. The simple introduction into its alimentary canal of large quantities of ferinaceous and gaseous material is not necessarily followed by a corresponding increase of developement. In all cases in which the food of an infant is said to be insufficient, the stools should be carefully examined, and if there be found in them the hard whitish or cheesy lumps, so characteristic of coagulated casein, it will be strong evidence that too much rather than too little is being given.

It is probably true that, in spite of all possible precaution, some infants will now and then be found with whom cow's milk will not agree. But before making any change, we should satisfy ourselves that it is the milk which is at fault, and not its mode of preparation.

Cow's milk may be be rendered more digestible by the addition of some alkali; lime-water is perhaps preferable. As lime-water contains only about one-half of a grain of lime to the ounce of water, it may be largely used in the dilution of the milk. This will in a great measure prevent the formation of those firm coagula of casein so difficult for the infant to digest.

It may not be uninteresting to pass in review some of the inevitable evils which follow the administration of improper food to infants. Irritation of the digestive organs will necessarily follow. Vomiting is speedily excited and the food rejected. This hint which Nature gives is too often disregarded by those to whose charge they are committed. Food of the same kind is given again and again, and soon the stomach loses in a measure its excitability, permitting a part or the whole of it to pass through undigested. The intestines then become irritated, and in their effort to get rid of these undigested matters, diarrhœa is excited. The evacuations are often horribly offensive—due to the decomposition or putrefaction which these matters have undergone. Such an infant would necessarily be expected to lose both flesh and strength; for, besides the weakness resulting from imperfect nutrition, there is the additional cause of debility from the repeated attacks of vomiting and purging, until soon its digestive powers are rendered so feeble that it is less than ever able to obtain any nourishment, from the diet with which it is furnished. A child thus erroneously fed often has a voracious appetite, which should be interpreted to mean that the ultimate structures of its economy are not satisfied. The quantity of food that it will sometimes swallow is enormous, and it is a matter of surprise to its attendants that, in spite of all this, wasting continues. It becomes peevish, fretful and irritable; when awake, it will cry almost incessantly from hunger, and then again from abdominal pains; and, to add to its suffering, it is also frequently attacked with obstinate cutaneous eruptions. That affection of the mouth known as thrush is exceedingly common, and when it occurs in an infant greatly reduced by a long course of improper feeding, betokens a condition of the digestive organs not at all favorable to the ready digestion and assimilation of food. If an infant of this description, with all power of endurance starved out of him, be overtaken by almost any acute disease, he will fall a ready victim to that which would be but a trivial ailment for a healthy child. Or if he escapes death from this cause, and continues to have supplied to him food which he cannot digest, the result will be the same, only a little longer deferred, as if all food were withheld. He will die of inanition. Or, if able to digest only a part of what is furnished him, his life may linger on, extreme emaciation attends him, his face bears the expression of age, his belly grows large, tubercles may or may not become developed, and finally, skin and bones, he sinks and dies. A certificate is given of death from marasmus. Or, if the infant be injudiciously fed from his very birth, his miserable existence will be brought to a more speedy end, for such an infant rarely lives longer than three months. He will become so weakened by the vomiting and diarrhœa ensuing upon a bad state of nutrition, as to be unable to make sufficient inspiratory effort to fill his lungs, and will die of sheer debility. Or, again, if he be overtaken by the warm weather of the summer season, cholera infantum will almost certainly end his existence.

It would be well were the evils of improper feeding confined alone to that class of unfortunate beings that are by necessity deprived of the breast.

Many an infant, with a full fountain of milk from which to draw its supply, is needlessly and wantonly stuffed with articles of food not only useless, but positively hurtful, through some mistaken notion or capricious longing for a fat baby on the part of its mother or nurse. It is not uncommon to see children at six months, and at times much younger, taken to the table with the family, and fed upon a promiscuous diet. This is a fruitful cause of diarrhœa, convulsions, and a great variety of other diseases among children.

By way of conclusion I would add, that as small things portend the danger in the artificial feeding of infants, so do small things go far towards warding it off. A word of advice in time will have the effect of staying death from many a home.—[From an Article in Med. Times, by W. M. Welch, M. D.

